# Enhancing the Opportunistic Bone Status Assessment Using Radiomics Based on Dual-Energy Spectral CT Material Decomposition Images

**DOI:** 10.3390/bioengineering11121257

**Published:** 2024-12-12

**Authors:** Qiye Cheng, Jingyi Zhang, Mengting Hu, Shigeng Wang, Yijun Liu, Jianying Li, Wei Wei

**Affiliations:** 1 Department of Radiology, First Affiliated Hospital of Dalian Medical University, Dalian 116000, China; cqy13469258368@163.com (Q.C.); jingyizhang0330@163.com (J.Z.); tttop_0819@163.com (M.H.); wangshigeng9855@163.com (S.W.); yijunliu1965@126.com (Y.L.); 2 CT Research, GE Healthcare, Dalian 116000, China; jianying.li@med.ge.com

**Keywords:** lumbar vertebrae, bone mineral density, material decomposition, spectral CT, radiomics

## Abstract

The dual-energy spectral CT (DEsCT) employs material decomposition (MD) technology, opening up novel avenues for the opportunistic assessment of bone status. Radiomics, a powerful tool for elucidating the structural and textural characteristics of bone, aids in the detection of mineral loss. Therefore, this study aims to compare the efficacy of bone status assessment using both bone density measurements and radiomics models derived from MD images and to further explore the clinical value of radiomics models. Methods: Retrospective data were collected from 307 patients who underwent both quantitative computed tomography (QCT) and full-abdomen DEsCT scans at our institution. Based on QCT measurements, patients were divided into three categories: normal bone mineral density (BMD), osteopenia, and osteoporosis. Using the abdominal DEsCT data, six types of MD images were reconstructed, including HAP (Water), HAP (Fat), Ca (Water), Ca (Fat), Fat (Ca), and Fat (HAP). Patients were randomly divided into a training cohort (*n* = 214) and a validation cohort (n = 93) at a ratio of 7:3. Focusing on the L1 to L3 vertebrae, density values from the six MD images were measured. Six density value models and six radiomics models were constructed using a random forest (RF) classifier. The performance of these models in assessing bone status was evaluated using the receiver operating characteristic (ROC) curves, and the DeLong test was employed to compare performance differences between the models. Results: The macro-area under the curve (AUC) values for the density value models based on HAP (Water), HAP (Fat), Ca (Water), and Ca (Fat) MD images were 0.870, 0.870, 0.847, and 0.765, respectively, which outperformed those of Fat (Ca) (AUC = 0.623) and Fat (HAP) (AUC = 0.618) density value models. In the comparison of radiomics models, the trends of model performance were consistent with the density value models across the six MD images. However, the models based on HAP (Water), Ca (Water), HAP (Fat), Ca (Fat), Fat (Ca), and Fat (HAP) images exhibited superior performance than those of the density value models with the corresponding MD images, with values of 0.946, 0.941, 0.934, 0.926, 0.831, and 0.824, respectively. Conclusions: Bone status assessment can be accurately conducted using density values from HAP (Water), HAP (Fat), Ca (Water), and Ca (Fat) MD images. However, radiomics models derived from MD images surpass traditional density measurement methods in evaluating bone status, highlighting their superior diagnostic potential.

## 1. Introduction

A 10% reduction in bone mineral density (BMD) can lead to a twofold increase in the risk of osteoporotic fractures [1]. These fractures may manifest in symptoms such as back pain and height loss, consequently exerting a substantial toll on the daily activities of affected patients [2]. Assessing bone status is fundamental in the early detection of patients with abnormal BMD and paves the way for preventive interventions aimed at curbing the incidence of osteoporotic fractures. Common techniques for measuring BMD include dual-energy X-ray absorptiometry (DXA) and quantitative computed tomography (QCT) [3]. DXA measures areal BMD (DXA-BMD) by analyzing the differential attenuation of high- and low-energy X-rays as they pass through bone and soft tissue, establishing it as the gold standard in diagnosing osteoporosis [4]. Nevertheless, the precision of DXA measurements can be compromised by lumbar degenerative osteoarthritis and abdominal aortic calcification, potentially leading to an overestimation of BMD [5]. QCT, a three-dimensional BMD measurement method, provides an independent quantification of trabecular bone density (QCT-BMD), which is more sensitive to changes in bone quality [6]. Compared to DXA-BMD, QCT-BMD is less susceptible to interference from adjacent tissues and offers greater spatial resolution. However, the broader clinical applicability of QCT is hindered by the requirement for specialized calibration phantoms and measurement software [7]. In light of this issue, experts proposed a more cost-effective approach to realize opportunistic bone status assessment using existing patient imaging data [8,9].

In recent years, dual-energy spectral CT (DEsCT) has emerged as a prominent modality in abdominal imaging, owing to its considerable benefits in enhancing image quality and improving lesion detection [10]. Utilizing existing DEsCT data for opportunistic bone status assessment can eliminate additional costs and radiation exposure associated with dedicated bone density measurements. In addition, virtual monochromatic imaging (VMI) and material decomposition (MD) represent key applications of DEsCT. VMI exploits material attenuation characteristics at discrete energy levels within each voxel to enhance image contrast, tailored to clinical needs [11,12]. However, VMI may struggle to differentiate between materials with analogous attenuation properties. In contrast, MD utilizes the attenuation differences at dual-energy levels to quantitatively evaluate specific materials, such as hydroxyapatite (HAP) and calcium (Ca) [13], which predominantly constitute the vertebra [14]. Choosing the optimal material combinations can more reliably represent the actual content of these materials within the target tissue. Consequently, further investigation is imperative to determine the most effective material pair for bone status assessment.

Radiomics excels in extracting substantial high-throughput quantitative features from medical images, encapsulating valuable insights and uncovering the heterogeneity of biological tissue [15]. Radiomics has been widely applied in diagnosing space-occupying lesions [16,17], evaluating therapeutic efficacy [18], and capturing intricate structural features in the trabecular bone associated with variations in bone density and strength [19]. Numerous studies have shown that binary classification radiomics models excelled in differentiating between non-osteoporotic and osteoporotic, or normal and abnormal BMD [20,21,22]. Nonetheless, developing a three-class radiomics model to categorize bone status into normal BMD, osteopenia, and osteoporosis offers a more comprehensive evaluation, thereby augmenting its clinical applicability for precise treatment.

Therefore, this study aims to identify the optimal combination of base materials in DEsCT and to compare the predictive performance in assessing bone status between radiomics models constructed from MD images and the traditional method based on density value measurements, evaluating the clinical utility of radiomics models in enhancing the efficacy and precision of bone status assessment.

## 2. Materials and Methods

### 2.1. Patients

**Figure 1:**
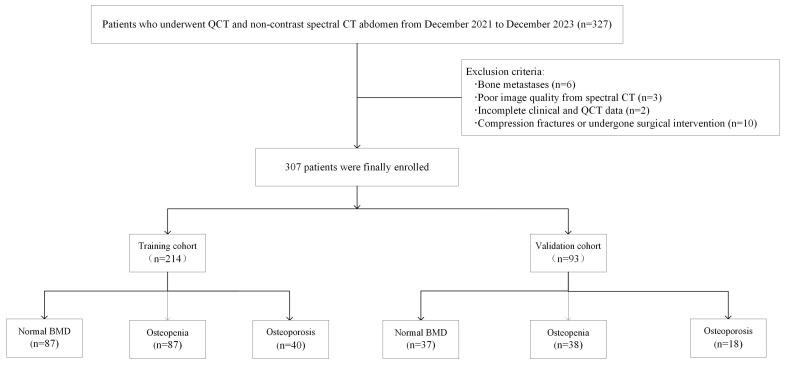
Flowchart of patient inclusion in this study.

This study received ethics approval from the institutional review board of our hospital. The informed consent was waived due to the retrospective nature of the study. A total of 328 patients who underwent both abdominal DEsCT and QCT examinations at our hospital within a week were retrospectively collected from December 2021 to January 2024. Patients were excluded if they had bone metastases, poor image quality, or incomplete clinical and QCT data. This study was targeted specifically at the L1, L2, and L3 vertebrae. Vertebrae were excluded if they presented with Grade 2 or more severe compression fractures, had a history of surgical intervention, or contained metal implants. The extent of vertebral compression was evaluated using the Genant classification [23]. The process for establishing inclusion and exclusion criteria is delineated in Figure 1. Basic patient demographics, including age, sex, body mass index (BMI), and bone status distribution, were recorded from the hospital case management system. Finally, a cohort of 307 patients was deemed eligible for this study. These patients were randomly divided into training and validation cohorts at a ratio of 7:3.

### 2.2. DEsCT Image Acquisition

**Figure 2:**
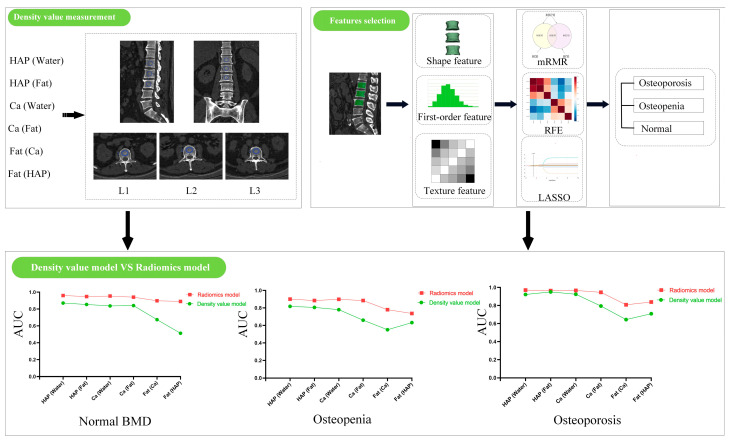
The overall pipeline of this study. mRMR, maximum relevance minimum redundancy; RFE, recursive feature elimination; LASSO, least absolute shrinkage and selection operator; BMD, bone mineral density. The AUC results are shown, with the green curve representing the density value model and the red curve corresponding to the radiomics model.

All patients underwent both QCT and abdominal DEsCT scans within one week using a 256-row CT scanner (Revolution CT, GE Healthcare, Milwaukee, WI, USA) at our hospital. The QCT scanning parameters were as follows: tube voltage of 120 kVp, Smart mA ranging from 100 to 600 mA. The abdominal DEsCT scanning parameters were as follows: Gemstone Spectral Imaging (GSI) mode with rapid switching between tube voltages of 80 kVp and 140 kVp, Smart mA ranging from 190 to 480 mA, and scan range from above the diaphragm to below the pubic symphysis. The remaining scanning parameters were kept the same: slice thickness of 5 mm, detector width of 80 mm, rotation time of 0.6 s/r, pitch of 0.992:1, and matrix of 512 × 512. Images were reconstructed with a slice thickness and interval of 1.25 mm to obtain the QCT images for measuring BMD, as well as HAP (Water), HAP (Fat), Ca (Water), Ca (Fat), Fat (HAP), and Fat (Ca) MD images for constructing density value models and radiomics models, as illustrated in Figure 2.

### 2.3. BMD Measurement in QCT

To ensure the precise measurement of BMD for the L1–L3 vertebrae, a calibration phantom (Model 4, Mindways Software, Inc., Austin, TX, USA) was scanned daily using the same 256-row CT scanner according to the QCT standard protocol before each QCT scan. Subsequent to the scan, calibration was performed on the QCT post-processing workstation, and the vertebral BMD was measured using QCT Pro software (version 6.1, Mindways Software, Inc., Austin, TX, USA) by a radiologist with 3 years of experience in diagnosing musculoskeletal disease. The measurement technique involves delineating a region of interest (ROI) on the central slice of the L1–L3 vertebrae, encapsulating roughly two-thirds of the trabecular bone area, while meticulously avoiding structures such as bone islands, osteophytes, cortical bone, lumbar facet joints, and the central vertebral canal. An automated process generates a high-resolution 9 mm volume of interest (VOI), which is fine-tuned in the sagittal plane to prevent it from crossing the cortical bone limits. Individual BMD values for each lumbar vertebra are documented in milligrams per cubic centimeter (mg/cm^3^), with the mean value from the three vertebrae serving as the definitive outcome. In line with guidelines set forth by the International Society for the American College of Radiology (ACR, 2013) [24], a BMD of less than 80 mg/cm^3^ is classified as osteoporosis, a BMD between 80 mg/cm^3^ and 120 mg/cm^3^ is classified as osteopenia, and a BMD greater than 120 mg/cm^3^ is classified as normal BMD.

### 2.4. Density Value Measurement and Model Construction

Density values for the six MD images were measured on a DEsCT system using Advantage Workstation 4.7 (AW4.7, GE Healthcare, Milwaukee, WI, USA) with dual-energy rapid switching at 80/140 kVp. The specific absorption characteristics of different substances to X-rays, representing tissue attenuation through two distinct material bases, enable quantitative material analysis. AW4.7 workstation software (AW4.7, GE Healthcare, Milwaukee, WI, USA) offers a variety of material base options, including HAP, Ca, water, and fat. By selecting any two material bases for MD imaging, DEsCT can effectively separate and relatively quantify target substances. In this study, HAP density was measured from HAP (Water) and HAP (Fat) images, Ca density from Ca (Water) and Ca (Fat) images, and Fat density from Fat (Ca) and Fat (HAP) images, with all density values expressed in mg/cm^3^. For each L1–L3 vertebra, an ROI was meticulously delineated within a region of uniform density, encompassing approximately two-thirds of the trabecular bone, while avoiding the venous plexus and bone islands. Density measurements were obtained from three consecutive axial slices for each vertebra, and their average values were adopted as the final outcome. Utilizing the random forest (RF) classifier, adapted from the scikit-learn package in Python 3.10 [25], density value models were developed based on six different MD images from the training cohort. To evaluate the model performance in predicting normal BMD, osteopenia, and osteoporosis within the validation cohort, receiver operating characteristic (ROC) curves were generated, and the area under the curve (AUC) values were calculated. For the three-class classification task, the macro-AUC approach was employed to compute the average AUC across all classification categories, providing a comprehensive evaluation of the overall classification performance. The evaluating indices, such as sensitivity, specificity, accuracy, and precision, were recorded to assess the predictive capabilities of each model on the validation set.

### 2.5. Radiomics Model Construction

#### 2.5.1. Image Segmentation and Feature Extraction

Six different MD images were imported into the uAI Research Portal (uRP: https://www.uii-ai.com/research.html?index=2, URL accessed on 15 August 2024) for the delineation of trabecular bone from L1 to L3 vertebrae. Following the delineation of the ROIs [26], a radiologist with 3 years of experience in diagnosing musculoskeletal disease manually refined the boundaries to exclude bone islands, osteophytes, cortical bone, lumbar facet joints, and the central groove of the vertebral body. One month later, a subset of 100 image sets from each MD image type was randomly selected for independent review by another radiologist with 23 years of experience in musculoskeletal diagnostics. The agreement between the ROI delineations by the two radiologists was assessed using the Dice similarity coefficient (DSC) and volume difference (VD). The DSC quantifies the proportional overlap between the two radiologists’ delineations, with a value of 1 indicating perfect concordance. The VD calculates the relative discrepancy in volume between these delineated regions.

Prior to feature extraction, the image data underwent a series of preprocessing steps. This included B-spline interpolation for image resampling, resulting in an isotropic resolution of 1 mm × 1 mm × 1 mm to ensure spatial uniformity and mitigate resolution-related errors. Subsequently, gray-level discretization with a bin width of 25 was applied to precisely capture the grayscale distribution characteristics of the image. To address variations in feature dimensions, Z-score normalization was implemented to standardize voxel values to a mean of 0 and a standard deviation of 1, thereby converting each voxel value into a standard normal distribution by subtracting the mean and dividing by the standard deviation (SD) of the overall voxel values, enhancing the stability and consistency of feature extraction.

After preprocessing, radiomics features were extracted from each ROI, including original features, as well as higher-order features processed with wavelet and Laplacian of Gaussian (LoG) filters (uAI Research Portal V1.1, United Imaging Intelligence, Co., Ltd., Shanghai, China). These processing steps were designed to capture subtle structural details and texture characteristics within the images. Three common groups of features were extracted, including shape features, first-order features, and texture features. Shape features pertain to the geometric structure and morphological characteristics of objects, including contour, volume, and surface morphology. First-order features provide a quantitative description of the pixel value distribution, offering insights into the overall gray-level characteristics. Texture features, involving the gray-level dependence matrix (GLDM), gray-level co-occurrence matrix (GLCM), gray-level run-length matrix (GLRLM), gray-level size-zone matrix (GLSZM), and neighborhood gray-tone difference matrix (NGTDM), are responsible for identifying patterns and variations in gray-level intensities, reflecting image roughness, texture orientation, and fine textural variations.

#### 2.5.2. Feature Screening and Models Construction

Feature selection and radiomics model construction were conducted using the uAI Research Portal, with the open-source Python package PyRadiomics employed for these processes. Initially, feature selection was performed using the maximum relevance minimum redundancy (mRMR) method, aiming to select the most predictive features by maximizing their relevance to the target variable while minimizing inter-feature redundancy. Subsequently, feature selection was further refined using the support vector machine (SVM) algorithm in combination with recursive feature elimination (RFE). SVM provides robust classification capabilities, while RFE optimizes the feature subset by recursively eliminating the least contributive features, thereby improving classification accuracy and mitigating the risk of overfitting. Finally, the optimized features were selected using the least absolute shrinkage and selection operator (LASSO) algorithm. LASSO employs L1 regularization to simplify the model by diminishing the weights of less critical features, thus reducing model reliance on less significant features.

With the optimized feature set established, six RF models were developed. The ROC curves were generated to evaluate the classification performance of these models. The effectiveness of the classification was quantified by calculating the AUCs.

### 2.6. Statistical Analysis

Statistical analysis was performed using SPSS version 26.0 (IBM Corp., Armonk, NY, USA) and MedCalc version 20.022 (MedCalc Ltd., Ostend, Belgium) software. Normality tests were conducted on continuous data. For normally distributed data, results were presented as mean ± SD, and group comparisons were made using one-way ANOVA. For abnormally distributed data, results were expressed as a median and interquartile range, with group comparisons performed using the Mann–Whitney U test. Categorical data were reported as frequencies and compared between groups using the chi-square test. The differences in AUC values were evaluated using the DeLong test. A significance level of *p* < 0.05 was adopted.

## 3. Results

### 3.1. Patient Demographics

The sex, age, BMI, and six density values of the 307 patients are detailed in Table 1 and Table 2. No statistically significant differences were observed in sex, age, or BMI across the three categories of bone status. However, significant differences were noted in the six material density values. The 307 patients were randomly divided into a training cohort (n = 214) and a validation cohort (n = 93) for model construction and validation. Comparisons within the training and validation cohorts across the three categories of bone status revealed statistically significant differences in sex and age.

### 3.2. Performance of the Density Measurement Value Models

Based on the mean density measurement values of HAP (Water), HAP (Fat), Ca (Water), Ca (Fat), Fat (Ca), and Fat (HAP) for the L1 to L3 vertebrae, optimal cutoff thresholds were determined through ROC curve analysis to enhance the sensitivity and specificity in predicting normal BMD, osteopenia, and osteoporosis. For the three-class classification of bone status, the macro-AUC values were 0.870 for both the HAP (Water) and HAP (Fat) models, 0.847 for the Ca (Water) model, and 0.765 for the Ca (Fat) model, surpassing those for the Fat (Ca) and Fat (HAP) models, which yielded macro-AUCs of 0.623 and 0.618, respectively (Table 3).

### 3.3. Performance of Radiomics Models

**Figure 3:**
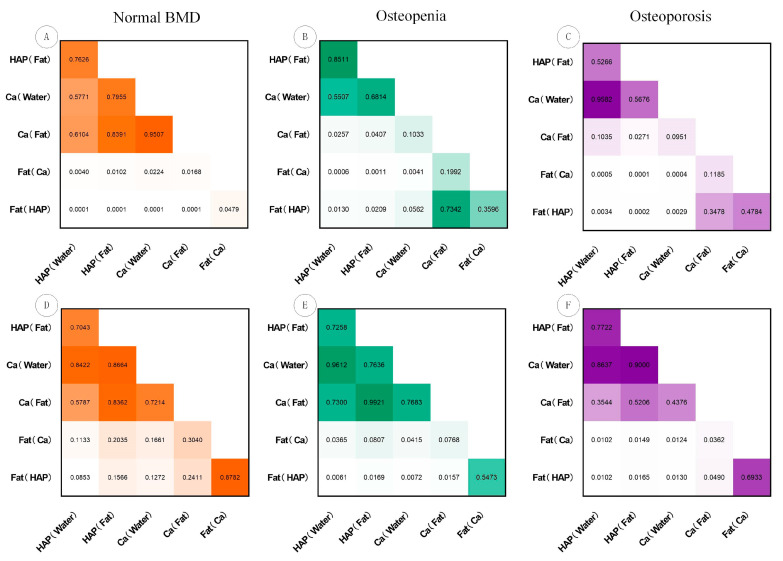
DeLong test results used to compare the diagnostic performance between the density value models and radiomics models constructed based on six MD images for predicting normal BMD, osteopenia, and osteoporosis within the validation cohort. (**A**–**C**) Density value models; (**D**–**F**) radiomics models.

The six MD images exhibited high consistency, with a mean DSC of 0.98 for defining the delineated ROIs (Table 4). The final number of features for the MD images of HAP (Water), HAP (Fat), Ca (Water), Ca (Fat), Fat (Ca), and Fat (HAP) was 6, 4, 5, 3, 7, and 7, respectively (Supplementary Table S1). The radiomics models developed with these refined features demonstrated strong efficacy in assessing the three categories of bone status. The macro-AUC values for the radiomics models based on MD images of HAP (Water), Ca (Water), HAP (Fat), and Ca (Fat) were 0.946, 0.941, 0.934, and 0.926, respectively, indicating a decreasing trend. These values outperformed those of the models derived from the Fat (Ca) and Fat (HAP) images, which had macro-AUC values of 0.831 and 0.824, respectively. The DeLong test results revealed that radiomics models constructed from HAP (Water), Ca (Water), HAP (Fat), and Ca (Fat) MD images demonstrated superior performance in predicting osteoporosis compared to the models based on Fat (Ca) and Fat (HAP) MD images (Figure 3).

### 3.4. Comparison Between Density Value Models and Radiomics Models

**Figure 4:**
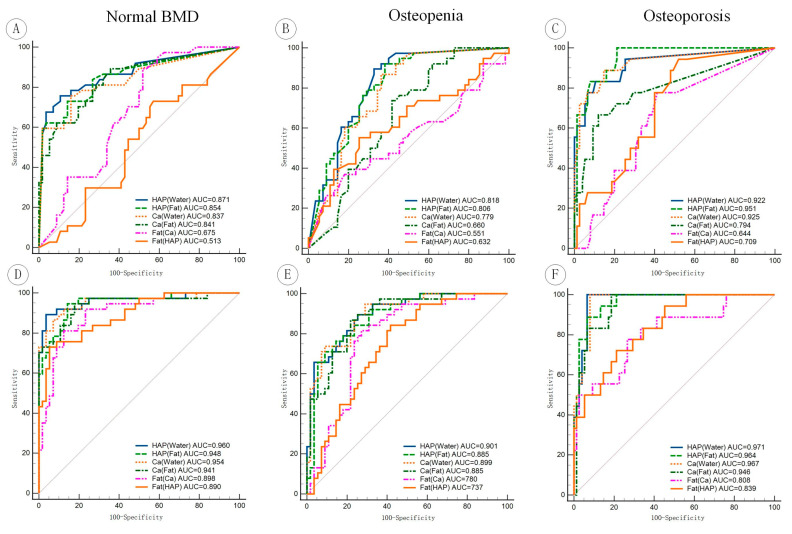
Comparison of ROC curves between different models in predicting normal BMD, osteopenia, and osteoporosis within the validation cohort. (**A**–**C**) Density values models; (**D**–**F**) radiomics models.

When comparing model performance under the same MD images, density value models consistently demonstrated inferior performance in the comprehensive assessment of bone status compared to radiomics models (Figure 4). Although the performance differences were statistically insignificant between the density value models and radiomics models based on HAP (Water), Ca (Water), HAP (Fat), and Ca (Fat) images in predicting normal BMD and osteoporosis, the radiomics models exhibited significantly higher performance than density-based models in predicting osteopenia (Table 5).

## 4. Discussion

Osteoporotic fracture is the most common complication of osteoporosis, frequently leading to limited mobility and a marked deterioration in quality of life [27], underscoring the great significance of opportunistic bone status assessment in effectively mitigating the risk of osteoporotic fractures. In our study, we developed six models based on material density values from DEsCT, each corresponding to a single MD image: HAP (Water), HAP (Fat), Ca (Water), Ca (Fat), Fat (Ca), and Fat (HAP). Additionally, we developed six radiomics models based on these MD images to assess bone status by extracting features from these MD images. The results indicated that radiomics models exhibited superior performance in bone status assessment compared to the simple density measurement value models derived from the same MD images. Notably, among the radiomics models based on MD images of HAP (Water), Ca (Water), HAP (Fat), and Ca (Fat), the macro-AUC gradually decreased, with values of 0.946, 0.934, 0.941, and 0.926, respectively, but all surpassed the performance of radiomics models based on Fat (Ca) and Fat (HAP) MD images yielding macro-AUC values of 0.831 and 0.824, respectively.

In alignment with consensus recommendations for QCT, the L1 and L2 vertebrae are identified as the standard and optimal sites for BMD measurement [28]. Nevertheless, the L3 vertebra is particularly sensitive to changes in bone quality [29] and can provide additional insights into bone loss [30]. Consequently, our study selected the L1 to L3 vertebrae for QCT-BMD measurements. Previous research [31,32,33] has demonstrated that the RF classifier is the most effective model for assessing vertebral bone status. To ensure consistency, the same vertebrae (L1 to L3) and RF classifier were utilized for constructing density value and radiomics models based on the six sets of MD images. The MD technique in DEsCT allows for the differentiation of various components within the vertebrae and enables their quantitative assessment [13]. Research by Wang et al. [34] and Yue et al. [35] demonstrated a robust correlation between the density values of HAP and Ca, obtained from MD images and QCT-BMD, with correlation coefficients of 0.912 and 0.851, respectively. Furthermore, Naik et al. [36] highlighted the potential of fat content, as measured by MRI Dixon sequences, in reflecting bone status. Guided by these findings, our study selected HAP, Ca, and fat as reference materials to form various base material pairs and analyzed the MD images of HAP (Fat), Ca (Fat), Fat (Ca), and Fat (HAP). Although water content also changes as a result of bone loss, these changes are primarily observed in the context of vertebral fractures [37]. Therefore, our study did not analyze water density values or water-based images. However, HAP (Water) and Ca (Water) MD images were reconstructed to determine whether density values for HAP and Ca with a fat background would outperform the performance of models using water as the background in bone status assessment.

Categorizing continuous variables, such as BMD, facilitates data interpretation and improves clinical decision making [38]. Typically, bone status is classified into three categories: normal BMD, osteopenia, and osteoporosis. Research has previously shown that the density values derived from MD images were proficient in differentiating between binary classifications of bone status [39,40]. In our study, three-class density value models were constructed to offer a more comprehensive assessment of bone status, thereby improving its clinical applicability. The results revealed that the density value models constructed using HAP-based images (HAP (Water) and HAP (Fat)) and Ca-based images (Ca (Water) and Ca (Fat)) achieved macro-AUC values of 0.870, 0.870, 0.847, and 0.765, respectively, which were significantly higher than those of the models based on fat-based images (Fat (Ca) and Fat (HAP)), with AUCs of 0.623 and 0.618, respectively. This discrepancy could be attributed to the more gradual changes in fat content during bone loss [41], whereas alterations in HAP and Ca content had a more direct and pronounced effect on bone density and structures.

Our study outcomes indicated that the performance trends of the radiomics models were in harmony with those of the density models. Specifically, radiomics models developed from HAP-based images and Ca-based images exhibited superior performance compared to those relying on fat-based images. As radiomics models make classification decisions based on features extracted from medical images, our analysis of the features involved in model construction revealed that first-order mean features were integral to the development of the four superior radiomics models using HAP-based and Ca-based images, while these features were notably absent in the models based on Fat-based images. The first-order mean feature represents the arithmetic mean of all voxel gray values, reflecting the average density within the image, which played an essential role in predicting bone status [22].

In the development of radiomics models, the filter was an essential aspect of image preprocessing, utilized to enhance specific structures and patterns within the images [42]. Wang et al. [22] constructed a radiomics model based on 70 keV images to diagnose osteoporosis, in which the original first-order mean feature made the greatest contribution, resulting in an AUC of 0.902. However, the AUC of this model was inferior to the radiomics models in our study, which were based on four MD images and achieved AUC values all exceeding 0.946 for differentiating osteoporosis. Further analysis indicated that all first-order mean features in our four models underwent log filter processing, which effectively highlighted discontinuities and sharp edges in the images [42]. This observation suggested that applying log filters to HAP-based and Ca-based images might enhance the performance of radiomics models in assessing bone status. Interestingly, HAP-based radiomics models consistently outperformed Ca-based models, regardless of whether water or fat was used as the background material. Specifically, the HAP (Water)-based model performed better than the Ca (Water)-based model, and the HAP (Fat)-based model performed better than the Ca (Fat)-based model, with macro-AUC values of 0.946, 0.941, 0.934, and 0.926, respectively. Although calcium is a vital component of bone composition, changes in bone density were also influenced by other inorganic components and tissue microstructures. HAP, serving as the primary inorganic bone component, possesses a stable bone mineral crystal structure that more accurately reflects bone density and detailed structural status [43], thereby providing higher sensitivity and specificity in bone status assessment. Additionally, our study found that isolating the water component for assessing HAP or Ca content in the vertebrae was more effective than using fat. In other words, the HAP (Water)-based model outperformed the HAP (Fat)-based model, and the Ca (Water)-based model performed better than the Ca (Fat)-based model. This is likely due to the more uniform distribution of water and its steady changes compared to fat during bone loss [44]. Consequently, the MD techniques of spectral CT enable a more accurate separation of water components, thereby enhancing the precision of HAP content measurement.

Density values derived from MD images reflect the relative content of specific bone components but are limited to one-dimensional analysis. In contrast, radiomics facilitates the quantitative extraction and examination of multidimensional imaging features, highlighting the diversity and complexity of bone detail architecture [45]. Jiang et al. [21] found that using Hounsfield Unit (HU) values of the lumbar spine from 120 kVp images was less effective in screening osteoporosis than radiomics models. Similarly, our study underscored the superior accuracy of radiomics models in predicting normal BMD, osteopenia, and osteoporosis, particularly in predicting osteopenia. The early identification of osteopenia facilitates timely intervention, with the potential to prevent the progression of osteoporosis and reduce the risk of fractures [30]. Despite the complexity of constructing radiomics models compared to direct density measurements, radiomics models can provide significant advantages in extracting intricate, detailed structural information from CT images, showing their broader potential for clinical applications.

Our study has several limitations that should be acknowledged. Firstly, as a retrospective study, this research may be susceptible to potential selection bias. Secondly, the relatively small sample size limits the generalizability of the results. Future research should validate these findings using larger, multi-center cohorts. Furthermore, despite our efforts to ensure that QCT-BMD and density values on MD images were measured on the same level, precise level matching between the two measurement methods was not achieved. Although QCT-BMD is widely recognized as a standard method for bone status assessment, its susceptibility to variations caused by bone marrow fat content may impact the accuracy of measurements and potentially affect the interpretation of the study results. Finally, our study only employed the RF classifier in constructing radiomics models, and the comparison of different machine learning algorithms should be considered in future research.

## 5. Conclusions

In conclusion, although conventional density value measurements on HAP (Water)-, Ca (Water)-, HAP (Fat)-, and Ca (Fat)-based MD images can assess bone status, radiomics models provide insights into intricate details of the trabecular network through textural features, introducing a more precise and holistic evaluation of bone status across the three diagnostic categories. Therefore, the integration of radiomics with MD techniques represents a promising, accurate, and innovative approach to opportunistic bone status assessment.

## Figures and Tables

**Table 1:** Clinical characteristics in the training and validation cohorts.

Variables	Training Cohort				Validation Cohort				
Bone Status	Normal BMD	Osteopenia	Osteoporosis	*p* Value	Normal BMD	Osteopenia	Osteoporosis	*p* Value	*p* Value *
	(*n* = 87)	(*n* = 87)	(*n* = 40)		(*n* = 37)	(*n* = 38)	(*n* = 18)		
Gender (n)									0.674
Female	26	35	25	0.002	14	9	12	0.008	
Male	61	52	15		23	29	6		
Age (years)	57.2 ± 11.4	64.5 ± 11.0	73.5 ± 6.9	<0.001	57.0 ± 10.1	65.1 ± 8.1	69.1 ± 7.6	<0.001	0.510
BMI (kg/m^2^)	24.2 ± 3.2	23.6 ± 3.1	23.6 ± 3.4	0.331	23.9 ± 3.5	24.0 ± 2.4	22.8 ± 2.8	0.302	0.763

* Significant difference between training and validation cohorts. BMD bone mineral density, BMI body mass index. Age and BMI are presented as mean ± standard deviation.

**Table 2:** Six density values in the training and validation cohorts.

Variables	Training Cohort				Testing Cohort			
Bone Status	Normal	Osteopenia	Osteoporosis	*p* Value	Normal	Osteopenia	Osteoporosis	*p* Value
HAP (Water) (mg/cm^3^)	112.7 ± 27.4	78.4 ± 14.2	51.8 ± 17.6	<0.001	111.3 ± 23.9	78.8 ± 14.2	49.3 ± 16.9	<0.001
HAP (Fat) (mg/cm^3^)	149.9 ± 28.2	115.7 ± 14.1	89.0 ± 17.6	<0.001	147.9 ± 24.4	116.0 ± 14.4	87.1 ± 16.7	<0.001
Ca (Water) (mg/cm^3^)	52.8 ± 12.8	36.7 ± 6.7	24.1 ± 8.2	<0.001	51.6 ± 11.6	36.8 ± 6.7	23.2 ± 8.1	<0.001
Ca (Fat) (mg/cm^3^)	71.3 ± 13.0	55.4 ± 7.9	42.3 ± 10.1	<0.001	70.6 ± 11.7	55.8 ± 7.0	43.7 ± 5.7	<0.001
Fat (Ca) (mg/cm^3^)	1015.0 ± 17.8	1001.4 ± 13.7	991.3 ± 19.2	<0.001	1018.2 ± 14.9	1000.2 ± 28.2	989.7 ± 12.1	<0.001
Fat (HAP) (mg/cm^3^)	952.5 ± 11.9	947.6 ± 9.3	942.7 ± 11.6	<0.001	951.0 ± 10.4	944.8 ± 8.9	942.9 ± 14.4	0.011

BMD bone mineral density, HAP hydroxyapatite, Ca calcium. Density values are presented as mean ± standard deviation.

**Table 3:** Comparison of diagnostic performance between density value model and radiomics models.

MD Images	Models	Macro-AUC	Sensitivity (%)	Specificity (%)	Accuracy (%)	Precision (%)
HAP (Water)	Density value model	0.870	82.83	81.96	82.80	70.98
	Radiomics model	0.946	74.50	88.40	78.50	80.50
HAP (Fat)	Density value model	0.870	88.36	75.40	79.21	64.19
	Radiomics model	0.934	78.30	88.40	78.50	82.20
Ca (Water)	Density value model	0.847	83.80	77.63	79.93	65.73
	Radiomics model	0.941	74.50	87.90	77.40	79.30
Ca (Fat)	Density value model	0.765	76.51	70.16	74.19	58.10
	Radiomics model	0.926	73.60	87.30	76.30	77.90
Fat (Ca)	Density value model	0.623	67.94	62.9	63.44	47.56
	Radiomics model	0.831	69.10	85.60	73.10	72.60
Fat (HAP)	Density value model	0.618	74.23	55.13	59.50	45.37
	Radiomics model	0.824	64.40	83.70	71.00	80.90

AUC area under the curve, MD material decomposition, HAP hydroxyapatite, Ca calcium.

**Table 4:** The Dice similarity coefficient and volume difference between two radiologists.

MD Images	DSC	VD (cm^3^)
HAP (Water)	0.98 ± 0.01	0.33 ± 0.21
HAP (Fat)	0.98 ± 0.01	0.34 ± 0.18
Ca (Water)	0.98 ± 0.01	0.33 ± 0.18
Ca (Fat)	0.98 ± 0.01	0.32 ± 0.18
Fat (Ca)	0.98 ± 0.01	0.34 ± 0.19
Fat (HAP)	0.98 ± 0.01	0.36 ± 0.18

MD, material decomposition; DSC, Dice similarity coefficient; VD, volume difference; HAP, hydroxyapatite; Ca, calcium.

**Table 5:** The results of DeLong test in comparing the six density value models with the six radiomics models, based on the same MD images in predicting bone status.

Variables	HAP (Water)	HAP (Fat)	Ca (Water)	Ca (Fat)	Fat (Ca)	Fat (HAP)
Normal BMD	0.193	0.245	0.183	0.295	0.058	0.001
Osteopenia	<0.0001	<0.0001	<0.0001	<0.0001	<0.0001	0.052
Osteoporosis	0.514	0.593	0.508	0.991	0.805	<0.05

BMD, bone mineral density; MD, material decomposition; HAP, hydroxyapatite; Ca, calcium.

## Data Availability

The raw data supporting the conclusions of this article will be made available by the authors without undue reservation.

## Supplementary Materials

The following supporting information can be downloaded at: https://www.mdpi.com/article/10.3390/bioengineering11121257/s1, Table S1: The final selected features for radiomics model construction.
