# Supplementary material for: Enhancing the Opportunistic Bone Status Assessment Using Radiomics Based on Dual-Energy Spectral CT Material Decomposition Images
Source: Bioengineering (Basel). 2024 Dec 12;11(12):1257. doi: 10.3390/bioengineering11121257 (PMC11673124; doi:10.3390/bioengineering11121257)
Supplement: Supplementary file 1 [file bioengineering-11-01257-s001.zip › bioengineering-3309683-supplementary.pdf]

Table S1. The final selected features for radiomics model construction

Image	Filtering method	Feature class	Feature name	Coefficient
HAP (Water)	log-sigma-4-0-mm-3D	firstorder	Mean	0.150562569
	wavelet-LLH	firstorder	10Percentile	0.135899767
	original	shape	Flatness	0.076996416
	original	firstorder	Minimum	-0.001313145
	original	firstorder	Energy	-0.07550202
	original	firstorder	10Percentile	-0.160866156
HAP (Fat)	wavelet-LLH	firstorder	10Percentile	0.171751142
	log-sigma-4-0-mm-3D	firstorder	Mean	0.161268473
	original	firstorder	Minimum	-0.008875684
	original	firstorder	10Percentile	-0.2654045
Ca (Water)	wavelet-LLH	firstorder	10Percentile	0.19968684
	log-sigma-4-0-mm-3D	firstorder	Mean	0.145927221
	log-sigma-4-0-mm-3D	gldm	LargeDependenceHighGrayLevelEmphasis	0.10896039
	original	firstorder	Energy	-0.07827035
	original	firstorder	10Percentile	-0.218000636
Ca (Fat)	log-sigma-4-0-mm-3D	firstorder	Mean	0.130292431
	log-sigma-4-0-mm-3D	glrlm	RunEntropy	0.061084375
	wavelet-LLL	firstorder	Median	-0.40911898
Fat (Ca)	wavelet-LLH	firstorder	10Percentile	0.259515375
	log-sigma-4-0-mm-3D	firstorder	90Percentile	0.158469558
	original	shape	Elongation	0.08607917
	log-sigma-0-5-mm-3D	glcm	JointEnergy	-0.002007273
	original	firstorder	Minimum	-0.005913156
	wavelet-LLL	firstorder	10Percentile	-0.126889259
	wavelet-LLL	firstorder	Mean	-0.195182234
Fat (HAP)	log-sigma-2-0-mm-3D	glrlm	RunVariance	0.123185925
	original	shape	Flatness	0.07190466
	original	shape	Elongation	0.026533354
	log-sigma-1-0-mm-3D	gldm	DependenceNonUniformityNormalized	0.01920284
	log-sigma-2-0-mm-3D	firstorder	90Percentile	-0.08328866
	original	glcm	Imc1	-0.177143

	log-sigma-2-0-mm-3D	firstorder	InterquartileRange	-0.1841957
